# Stakeholders’ perspectives on training over the counter medicine sellers and Community-based Health Planning and Services facilities to dispense antibiotics in Ghana

**DOI:** 10.1186/s40545-021-00349-0

**Published:** 2021-07-22

**Authors:** Samuel Afari-Asiedu, Marlies Hulscher, Martha Ali Abdulai, Ellen Boamah-Kaali, Heiman F. L. Wertheim, Kwaku Poku Asante

**Affiliations:** 1 Kintampo Health Research Centre, Research and Development Division, Ghana Health Service , Kintampo, Bono East Region Ghana; 2 Radboudumc Center for Infectious Diseases , Radboud University Medical Center, Nijmegen, The Netherlands; 3 Radboud Institute for Health Sciences, Department of Medical Microbiology, Radboud University Medical Centre, Nijmegen, The Netherlands; 4 Scientific Center for Quality of Healthcare, Radboud University Medical Center, Nijmegen, The Netherlands

**Keywords:** Ghana, Antibiotic, Antibiotics sales, Stakeholders, Over the counter medicine sellers, Community-based Health Planning and Services

## Abstract

**Background:**

Dispensing of antibiotics by over the counter medicine sellers (OTCMS) is a major driver of inappropriate use and resistance in low and middle income countries. Recent studies in Ghana revealed the need to consider training OTCMS and Community-based Health Planning and Services (CHPS)/health posts to dispense some antibiotics. Feasibility of training OTCMS and CHPS to dispense some antibiotics was explored in this study.

**Methods:**

This was an explorative study involving 10 in-depth interviews (IDIs) among staff of Ghana health services (GHS), pharmacy council and the association of OTCMS at the district and regional levels. Next, findings were presented to the Ghana Antimicrobial Resistance (AMR) platform for further discussions at the national level. Five IDIs were also performed among selected members of the AMR platform as a follow-up on emerging issues. Data were thematically analysed and presented as narratives with quotes to support the findings.

**Results:**

Two opposing views were found in our study. Leadership of OTCMS and GHS staff at the district health directorate supported the suggestion that OTCMS and CHPS should be trained to dispense specific antibiotics because they are already dispensing them. The leadership of OTCMS explained that some of their members are experienced and could be trained to improve their practices. In contrast, participants from pharmacy council, GHS in the region and national AMR platform generally alluded that OTCMS and CHPS should not be trained to dispense antibiotics because their level of education is inadequate. GHS personnel from the region further explained that training OTCMS could further compromise inappropriate antibiotic use in the context of already weak regulation enforcement. GHS and pharmacy council in the region rather suggested that OTCMS and CHPS should focus on public health education on disease prevention and appropriate antibiotic use.

**Conclusions:**

There is general lack of consensus among stakeholders on whether OTCMS and CHPS should be trained to dispense specific antibiotics. Further stakeholder engagement is required to carefully consider this suggestion as views on feasibility differ. Ministries of health and healthcare agencies in Ghana and LMIC should improve access to approved health services to improve antibiotic use in rural settings.

## Background

The widespread availability and use of antibiotics are major drivers of resistance in low and middle income counties (LMIC) [1–3]. Inappropriate access and use are influenced by weak implementation of regulations regarding the sales of antibiotics, customer demands, distance to and delays in healthcare provision in approved health facilities and financial gains by over the counter medicine sellers (OTCMS) [4, 5]. Consequently, rural residents who are generally of low socioeconomic status often buy antibiotics from unapproved sources such as OTCMS and even drug peddlers who are closer to them [4–7]. Public community health posts (CHP) known in Ghana as Community-based Health Planning and Services (CHPS) facilities and OTCMS previously referred to as Licensed Chemical Sellers in Ghana are therefore important sources of healthcare provision especially in rural areas [4–7]. CHPS was adopted in 1999 as a national primary healthcare initiative with a network of health post at the community level. With a focus on preventive health services, activities of CHPS facilities mainly include health promotion, vital health event recording, community mobilization for health activities, disease surveillance and basic health service delivery [8, 9]. Recently, CHPS facilities with midwives are allowed to dispense some antibiotics as part of curative service provision.

In LMIC, antibiotics dispensing by OTCMS is a pharmaceutical regulatory issue which often creates tension between regulatory bodies and OTCMS [4]. Whilst pharmaceutical regulations in most LMIC prevent OTCMS from dispensing antibiotics, they sell antibiotics against regulations. In Ghana, though OTCMS by regulations are not permitted to sell/dispense antibiotics which is a prescription only medicine except cotrimoxazole, in practice they sell different types of antibiotics with or without prescription [4, 10]. The Health Professions Regulatory Body Act, 2013 (Act 857) which came to replace the Pharmacy Act, 1994 is the main law that regulate pharmacy practice in Ghana. Similar to the Pharmacy Act, 1994, Act 857 indicates that only doctors, physician assistants, midwives, pharmacist and nurse prescribers are eligible to prescribe and dispense registered antibiotics [11]. The Act 857 further prescribes sanctions for defaulting OTCMS including revoking their license and a fine not exceeding 250 penalty units. However, beyond the weak implementation of regulation regarding the sale of prescriptions only medicines including antibiotics, sanctions are barely applied by the Pharmacy Council hence the evasion of the regulation by OTCMS and drug peddlers [4].

Our recent studies in central Ghana revealed that training of OTCMS and CHPS facilities to dispense specific antibiotics could be one of the context-specific solutions to improving antibiotic access and use at the community level [4, 5]. For instance, OTCMS and CHPS could be trained to dispense access group antibiotics as they could treat a wide range of common bacterial infections and less likely to develop resistance compared to antibiotics in watch and reserve groups. WHO has categorized antibiotics into ACCESS, WATCH and RESERVE (AWaRe) groups to help in the development of tools for stewardship programmes and to reduce antimicrobial resistance [12, 13].

In our previous studies, we explored the perspective of antibiotic dispensers and community members on the regulatory and community demands on the sale of antibiotics; the views of policy-makers, health services administrators and regulators were not represented as the study focused on contextual factors at the community level [4, 5]. This study therefore sought to explore stakeholders’ perspectives on the feasibility of training OTCMS and CHPS facilities to dispense some antibiotics in Ghana.

## Methods

### Study design/approach

This was an explorative study involving in-depth interviews (IDIs) with stakeholders and guided by a bottom-up research approach. The bottom-up approach is a participatory design which aims at involving relevant stakeholders from grassroots through to policy-makers/management level in the process of ideation, consensus building, and policy formulation [14–16]. Unlike the top-down approach where actors at the top (management level) formulate policies, the bottom-up approach recognizes that actors who are connected with the actual situation could make more realistic inputs than the policy-makers who do not have the same information [16, 17]. This approach is important as top-down interventions by governments and relevant institutions targeting behaviour change at the community level may be unrealistic, impractical and undesirable, and may adversely affect access to healthcare [17].

In this study, we sought to bring to the attention of relevant stakeholders involved in pharmaceutical policy formulation and implementation in Ghana the findings which emerged from our previous study involving OTCMS and community members [4]. Bringing these findings to the attention of relevant stakeholders and exploring their perspective will contribute to designing interventions to improve antibiotic access and use at the community level. This study therefore started with IDIs among personnel of Ghana health services (GHS), leaders of OTCMS association at the district and regional levels. Following the IDIs, presentation of the preliminary findings were made on the Ghana National Antimicrobial Resistance (AMR) platform to further explore the perspective of policy-makers, health services administrators, regulators and other relevant stakeholders present at the meeting. A second round of IDIs were conducted among selected Ghana AMR platform members as a follow-up on some of the issues that emerged during the presentation and discussions. The focus was therefore on building a trajectory of moving what originated from the community level in our previous study for consideration in formulating policies/regulations and designing interventions.

### Application of a health system perspective

**Fig. 1:**
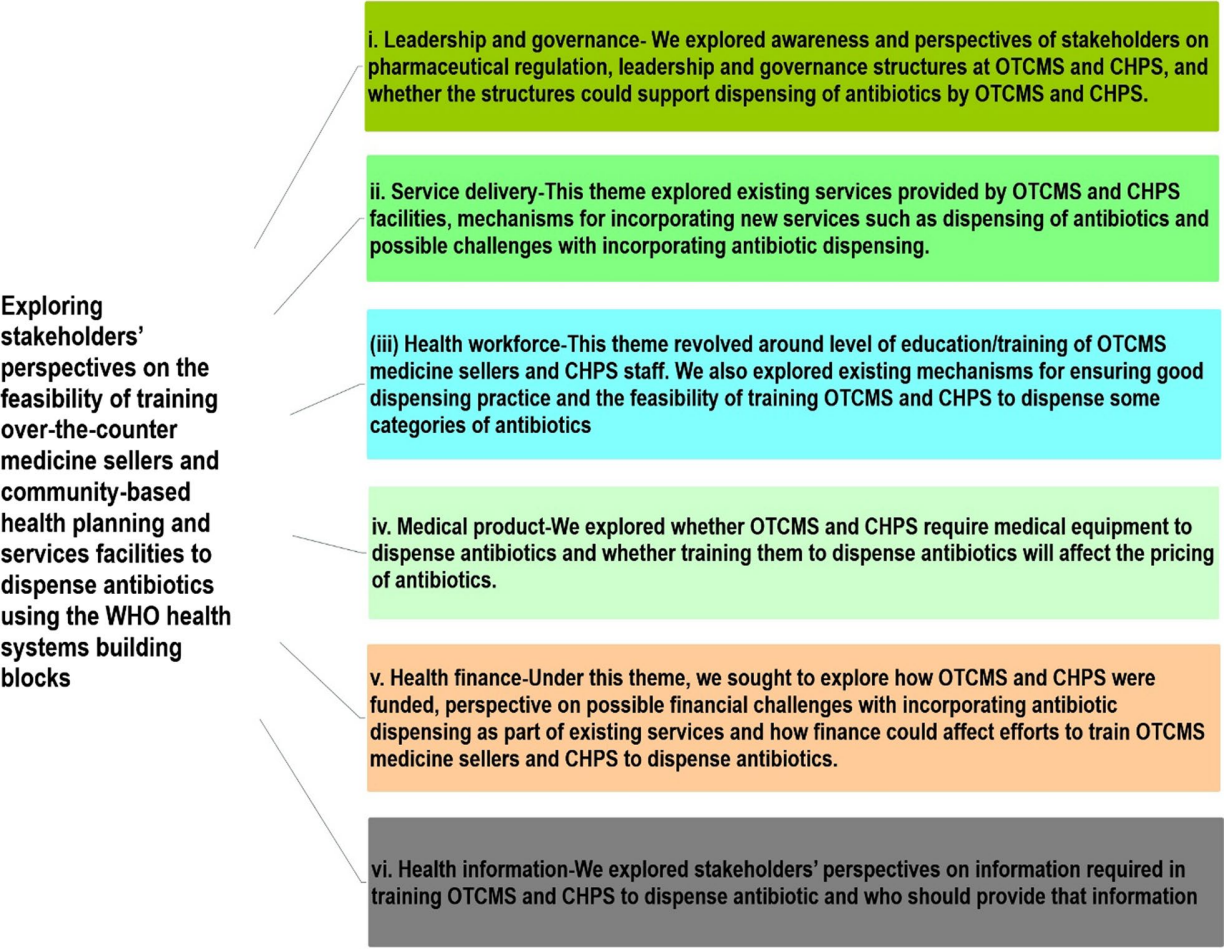
Summary of themes explored following the WHO building blocks

A health system perspective was applied in designing the study guides to explore stakeholders’ perspectives on the feasibility of allowing OTCMS medicine sellers and CHPS facilities to dispense some antibiotics. This was to ensure that all the aspects of a potential health system which allows the dispensing of antibiotics by OTCMS and CHPS facilities were covered during interviews with stakeholders/participants. The IDI guide for the district and regional stakeholders’ interviews was therefore organized around the WHO health system building blocks including leadership and governance, service delivery, health workforce, medicine and medical products, finance and health information (Fig. 1) [18, 19].

In Ghana, OTCMS and CHPS facilities are part of the health system and major sources—if not the first point of call—for healthcare when people are ill in the community. OTCMS and CHPS facilities by regulation have predefined services they are allowed to provide and this does not include dispensing of antibiotics. Allowing them to dispense antibiotic may require policy and regulatory reviews and this has health systems implications. For instance, allowing OTCMS and CHPS facilities to dispense antibiotics could affect prices of antibiotics which could influence health seeking behaviour, personnel may have to be trained and this requires funds. Consequently, the application of the WHO building blocks enabled us to broadly explore stakeholders’ perspectives on health system issues that could influence the suggestion of allowing OTCMS and CHPS to dispense some categories of antibiotic.

### Study area/context

This study was conducted among stakeholders in the Kintampo north municipality (KNM), Sunyani the Brong Ahafo (now divided into Bono, Ahafo and Bono east regions) regional capital and the national level in Accra. Ghana operates a centralized system of governance with 16 administrative regions. Governance is therefore decentralized along this structure; from district/municipality through the region to the national level and vice-versa. Stakeholders involved in pharmaceutical regulations also operate in line with this decentralized structure hence the need for stakeholders at all levels to be engaged for their perspectives on training OTCMS and CHPS facilities to dispense some categories of antibiotics. Interviews commenced at the district level in KNM, one of the two sites where the previous studies that explored the perspective of antibiotic suppliers and community members on access to and use of antibiotics were performed [4, 5]. The KNM is located within the forest–savannah transitional ecological zone in the Bono East Region and covers an area of 5108 km^2^ with a resident population of approximately 93,107 as at 2019 [20, 21]. The KNM is largely rural and the majority of inhabitants initiate treatment for some ailments at home, then continue to the OTCMS to buy medicines including antibiotics, and they may finally end up in a public health facility if their illnesses do not resolve. Most districts in the Bono East (part of the then Brong Ahafo) Region and Ghana share the health seeking behaviour of people in the KNM [22]. About 80% of medicine outlets in rural communities in Ghana are OTCMS, who are mostly the first point of contact for healthcare and a majority if not all of them dispense antibiotics against regulation [23].

### Data collection

#### IDIs at the district and regional levels

A total of 10 respondents were purposefully selected at district and regional level (Table 1). At the district level, IDIs were conducted among GHS personnel including the municipal director of health services, disease control officer, and a public health nurse. We also interviewed the vice chairman and secretary of OTCMS association in the district. At the regional level, interviews were conducted with the regional director and deputy regional director of GHS, the regional manager of the pharmacy council of Ghana as well as the deputy regional chairman and secretary of OTCMS association. Respondents were selected from these institutions because they are involved in formulating and ensuring compliance to pharmaceutical practice in Ghana (Table 3). Also, interviewing stakeholders from these institutions/association brought divergent perspectives to bear on how to improve access and use of antibiotics at the community level in Ghana.

**Table. 1:** Summary of IDIs conducted

Respondents	Institutions/agencies	No. of IDI
District		
Director of health services	Municipal Health Directorate-GHS, Kintampo North	1
Public Health Nurse	Municipal Health Directorate-GHS, Kintampo North	1
Disease control officer	Municipal Health Directorate-GHS, Kintampo North	1
District deputy chairman/OTCMS	OTCMS association, Kintampo North	1
District secretary/OTCMS	OTCMS association, Kintampo North	1
Region		
Director of health services	Brong Ahafo Regional Health Directorate-GHS	1
Deputy director of health services (clinical care)	Brong Ahafo Regional Health Directorate-GHS	1
Deputy Regional Manager	Pharmacy council, Brong Ahafo Region	1
Deputy Regional Chairman/OTCMS	OTCMS association, Brong Ahafo Region	1
Regional Secretary/OTCMS	OTCMS association, Brong Ahafo Region	1
National (Ghana AMR platform)		
Pharmacist/deputy chairman	Ghana AMR platform	1
Pharmacist/AMR Technical Officer, Member of AMR platform	WHO country Office, Ghana	1
Food microbiologist, Member of AMR platform	Ghana FDA, Member of AMR platform	1
Pharmacist/Rector, Member of AMR platform	Ghana College of Pharmacists	1
Pharmacist/vice president, Member of AMR platform	Pharmaceutical society of Ghana	1
Total		15

#### Presentation and discussions on the AMR platform and follow-up IDIs at the national level

The study took advantage of the Ghana AMR platform quarterly meetings to present preliminary findings from the district and regional IDIs and to discuss the feasibility of training OTCMS and CHPS facilities to dispense some antibiotics. The AMR platform was chosen for the stakeholder engagement at the national level because as indicated above, it constitutes all the relevant institutions which developed the National AMR policy and are involved in the implementation of various activities towards the containment of AMR in Ghana. It therefore served as a good platform to present and discuss the perspectives of these stakeholders.

Another round of IDIs was conducted among selected platform members as a follow-up on some of the issues that emerged during the presentation and discussions at the AMR platform meeting. Five (5) IDIs were conducted with a pharmacist/deputy chairman of the AMR platform, pharmacist/AMR Technical Officer at WHO country Office, Ghana, a food microbiologist at the Ghana Food Drugs Authority (FDA), the Rector of Ghana College of Pharmacists and a pharmacist at the pharmaceutical society of Ghana.

### Study procedures

#### IDIs at the district, regional and national levels

Participants were informed about the purpose and procedures of the study. Informed consent was read to the potential participants. Participants who agreed to take part in the IDIs at the district and regional levels were provided with copies of the written study information sheets, and signed consent forms which were collected before the actual interviews. Follow-up IDIs at the national level, with representatives of the AMR platform were conducted over telephone. Participants were sent emails to explain the purpose of the follow-up interviews and to seek for their consent to participate. Having been part of our previous engagement during the presentation and discussions of the preliminary findings on the Ghana AMR platform, participants agreed to be part of the study and to be recorded. All participants who were contacted agreed to be interviewed except the regional chief pharmacist who could not be interviewed due to time constraints as he was officially engaged during the data collection period. Interviews were audio recorded and conducted by a moderator and a note-taker (the PI and a team member who are trained Social Scientist). IDIs lasted for about 30–45 min and were conducted face-to-face in English or Twi (widely spoken local dialect in the study area). All sessions were brought to an end when the moderator had exhausted all questions in the interview/discussion guide and on other emerging issues.

#### Presentation and discussions on the AMR platform

As representatives of the Kintampo Health Research Centre (KHRC) on Ghana AMR platform, we informed/notified the secretary of our intention to present preliminary findings and further explore the perspective of representatives on the platform. Subsequently, an email was sent with an informed consent form to the secretary to further explain the purpose of the study and this was shared with platform members prior to the meeting. In consultation with the chairperson, the presentation and discussion was included in the agenda of the meeting. At the meeting the chairperson introduced the presenter (a team member who is trained in public health) who took the members at the meeting through the informed consent form including the fact that discussions after the presentation will be recorded. All AMR platform members in attendance verbally agreed to be part of the discussion and this was confirmed by the chairperson before the presentation started. The presentation was done in English for 15 min followed by 25 min discussion session moderated by the presenter with the help of the chairperson, and was audio recorded. In all, nine (9) representatives took part in active discussions.

### Data management and analysis

Audio recordings of IDIs including those conducted in Twi and discussions on the AMR platform were transcribed into English language verbatim. The transcripts were uploaded into qualitative data analysis software (NVivo 10) for coding and analysis by the study PI. In Nvivo, major and sub-themes were created in line with a priori themes (WHO health system building blocks) and responses were coded accordingly. Two team members (the PI and a team member who are trained in social science and public health) independently coded two transcripts. This was followed by a debriefing session to assess the level of consensuses and any disagreement was resolved through discussion. The remaining transcripts were coded by the PI. New themes that emerged during the coding were discussed with the initial independent coder and included as part of the coding frame. This was followed by an interpretive analysis of the collated responses.

## Results

The results section comprises six sub-sections following the WHO building blocks, including (i) leadership and governance; (ii) service delivery; (iii) health workforce; (iv) medicine and medical products; (v) health finance and (vi) health information. Results are presented as a narrative with selected quotes to support the findings (Table 2).

**Table. 2:** Summary of key findings

1. Leadership and governance	GHS at the district and OTCMS at the district and regional levels: leadership structure of OTCMS and CHPS can support the dispensing of antibiotics GHS and pharmacy council at regional and the national AMR platform: leadership and governance structure of OTCMS and CHPS cannot support the dispensing of antibiotics
2. Service delivery	OTCMS and GHS at the district level: OTCMS and CHPS could be trained to dispense antibiotic GHS, pharmacy council at the regional level and the national AMR platform: allowing OTCMS and CHPS to dispense antibiotics could further dilute the system
3. Health workforce	OTCMS at the district and regional level: it is possible to train OTCMS to dispense antibiotics as they have extensive years of experience GHS at the district level: it is feasible to train health workers in CHPS to dispense some antibiotics as they are already trained and doing similar activities GHS and pharmacy council at the regional levels and national AMR platform: OTCMS cannot be trained to dispense antibiotics because their level of education is inadequate
4. Medical products	GHS at the district and regional levels: medical laboratory which is currently not part of CHPS with basic equipment may be required to do culture and sensitivity test OTCMS at the regional level: there will be no need for such equipment as antibiotics would be dispensed with prescription from health professionals OTCMS in district and regional levels: prices of antibiotics may increase if they would have to buy equipment, i.e. rapid diagnostic test kits
5. Health finance	OTCMS at the district and regional levels: there would be no major financial challenges with dispensing antibiotics as OTCMS could buy medicines on credit from the pharmaceutical companies and pay later GHS and pharmacy council: financial implications—equipping and training staff could be challenging
6. Health information	OTCMS at the regional level: a manual/guideline with information on antibiotics dispensing will be required for reference GHS participants at the district level: Beyond antibiotics dispensing guidelines, health information materials such as posters and reporting format for antibiotics dispensed will be required

### Leadership and governance

#### Perspective on pharmaceutical regulation

There were two main perspectives on the current pharmaceutical regulation which prevents OTCMS and CHPS facilities from dispensing antibiotics. First, participants from GHS and pharmacy council at the regional level and Ghana AMR platform at the national level indicated that the current regulation is good and should be maintained. They emphasized that revising the current regulation will worsen inappropriate antibiotic access and use in the context of already weak implementation of regulation.

“…OTCMS are not supposed to be treating infections. Infections can be very tricky, you may not be sure of the infection you are managing, revising the regulations for them to dispense antibiotics may rather worsen the situation” (IDI, Ghana AMR platform, respondent #1).

There was however a dissent as a respondent at the regional level expressed uncertainty on whether the current regulation should be revised or not considering that unapproved medicine sellers are already dispensing antibiotics:

“…it’s not advisable to allow OTCMS and CHPS facilities to dispense antibiotics. However, looking at the situation…it will have a balancing effect if we train the OTCMS to at least give information on antibiotics to clients” (IDI, regional health directorates, GHS, respondent #2).

Also, participants of GHS at the district level and the leadership of OTCMS association at the district and regional levels were of the view that the current regulation should be revised. Leadership of OTCMS association at the regional levels specifically emphasized that the current regulation which prevents OTCMS from dispensing antibiotics has outlived its usefulness and should be revised to meet the demands of the growing population. According to them there is a limited number of pharmacies to serve the growing population especially in the rural areas.

“…the regulation has outlived its usefulness…we have over thousand OTCMS in the region whiles the pharmacies are not even up to hundred…we [OTCMS] are in the rural areas. It will be good if they upgrade our license, as the population is increasing” (IDI, OTCMS association, Brong Ahafo, respondent #2)

#### Leadership and governance structure at OTCMS and CHPS facilities

OTCMS are supervised/regulated by the pharmacy council of Ghana and are usually managed privately by the licensed holders with the support of an attendant or a family relative. The dynamics of leadership at CHPS is as explained in the excerpt below.

“In CHPS facilities, we have a midwife, a community health officer (CHO) who is a preventive nurse, enrolled nurse who is curative and in some instance a disease control officer; in that context the midwife is in-charge. This is however not the case in all CHPS because not all of them have midwives; where there is no midwife, the CHO becomes the head” (IDI, Municipal Health Directorate, Kintampo, respondent #2).

#### Whether leadership and governance of OTCMS and CHPS could support antibiotics dispensing

GHS participants at the district level indicated that the current leadership and governance structure of CHPS facilities could support the dispensing of antibiotics if the policy is revised. Similarly, leadership of OTCMS association at district and regional levels were of the view that with the support of their association and supervision by pharmacy council, the current structure could support the dispensing of antibiotics by OTCMS.

“Some staff at CHPS may have the competence to dispense antibiotics… It’s all about the guidelines, thus, there should be some modifications. We can start by giving them in-service training to dispense some basic antibiotics” IDI, Municipal Health Directorate, Kintampo, respondent #2).

In contrast, GHS participants at the regional level, pharmacy council and the AMR platform emphasized that the leadership and governance structure at OTCMS and CHPS cannot support the dispensing of antibiotics. The main challenge specified revolves around category of staff/workers who they indicate are not qualified enough and governance (policy issues), thus allowing OTCMS and CHPS to dispense antibiotics requires changes in policy and regulations.

The framers of the law intentionally categorized health staff who can dispense antibiotic; OTCMS have category of medicines they can dispense and obviously antibiotics is not one of them. It is just right from the policy perspective to say no. AMR issues are serious issues; very serious issues, as it is we are all at risk… I think this study is good but from where I sit it is emphatically no and from a policy perspective it is a no no no (Discussions on the AMR platform, respondent #9)

### Service delivery

#### Existing services provided by OTCMS and CHPS facilities

CHPS facilities provide both preventive and curative health services. The preventive health services include health promotion, immunization, home visiting, whilst curative service include treatment of minor ailments such as uncomplicated malaria, antennal, delivery and postnatal services. OTCMS are permitted to sell only OTC medicines excluding the sale of antibiotics except cotrimoxazole which is commonly dispensed for the treatment of infective diarrhoea, urinary tract infections and upper respiratory tract infections[4, 24].

#### Mechanisms for incorporating new services

According to the leadership of OTCMS association, introducing new services into existing ones were done previously in collaboration with the pharmacy council and OTCMS association. Specific reference was made to the pharmacy council which organizes refresher training once or twice a year for OTCMS. As such the pharmacy council will train them when the dispensing of antibiotic is included as part of the medicines they could dispense. Participants further cited examples of how they were trained to be able to test for malaria before dispensing antimalarial which hitherto was not part of the services they provided.

“Previously, we were not doing malaria test; it was KHRC in collaboration with the Ghana Malaria Control Programme and pharmacy council that trained us on testing for malaria with rapid diagnostic test” (IDI, OTCMS association, Brong Ahafo, respondent #1)

With regard to CHPS, GHS participants at the district level indicated that the health system is adaptable and allows new services to be added to existing ones when necessary and feasible. Reference was made to how some curative services have been included as part of services provided by CHPS which used to be preventive oriented.

“The system (CHPS) always allows new services to be incorporated if only it is feasible” (IDI, Municipal Health Directorate, Kintampo, respondent #2).

#### Possible challenges with incorporating antibiotics

GHS participants, pharmacy council at the regional level and the AMR platform emphasized profit which was the overarching reason for the sale of medicine by OTCMS and will be a major challenge when OTCMS are allowed to dispense antibiotics. They further stressed that OTCMS were not likely to follow the guidelines for antibiotic dispensing even if they are trained because of profit:

“You have a brother in the house telling you to go and buy amoxicillin; when the person walks to the pharmacy and the attendant is a pharmacist he will not sell it…. For the OTCMS, even if they are trained to understand the negative side effects of dispensing antibiotics they will still sell it for profit” (IDI, pharmacy council, Brong Ahafo, respondent #1).

Whilst the leadership of OTCMS medicine sellers association at the regional level foresee resistance from pharmacy owners as a possible challenge, they corroborated the finding that OTCMS are not likely to follow guideline when they are allowed to dispense antibiotics.

“…most of us [OTCMS] do not follow instructions. For instance if someone request for tetracycline; we have always been telling them not to sell half dosage; but because of money some ignore. (IDI, OTCMS association, Kintampo, respondent #2)

### Health workforce

#### Level of education/training of OTCMS medicine sellers and CHPS

The minimum level of education required to practice as an OTCMS is Senior High School certificate. It emerged that there was no specific training organized for the OTCMS by the pharmacy council before a licence is given. The requirement is that applicants of OTCMS licence should acquire/learn dispensing through attachment with an experienced OTCMS for a period of time (unspecified). Some applicants come in already trained as dispensary technician and pharmacy assistant. All applicants were provided with dispensing manuals by the pharmacy council to study for exams to test their dispensing knowledge. OTCMS who passed the exams were further interviewed on the dispensing knowledge and given orientation on do’s and don’ts, especially the types of medicines you can dispense before a licence is issued:

“There is no specific training for OTCMS. Currently; if someone want to be an OTCMS; the person can attach himself to me and learn from me” (IDI, OTCMS medicine sellers association, Kintampo, respondent #2).

At CHPS, the staff are mainly health workers of GHS with training in certificate or diploma. For instance, CHOs are awarded a certificate in community health nursing (CHN) following a 2-year training. After graduation, the CHNs receive 2 weeks orientation before they are finally posted to their respective CHPS facilities as CHO.

#### Existing mechanisms for ensuring good dispensing practice

The pharmacy council, FDA and pharmaceutical companies organize refresher training for OTCMS. Whilst the refresher training by the pharmacy council was organized for all OTCMS every year, training by FDA and pharmaceutical companies was conducted once in a while among selected OTCMS. Mechanisms of ensuring good dispensing practices at the CHPS include refresher training, continuous monitoring and supportive supervision.

#### Feasibility of training OTCMS and CHPS to dispense some categories of antibiotics

Leadership of OTCMS association indicated that it is possible to train OTCMS to dispense antibiotics considering the extensive years of experience of some of them in dispensing medicines. It was alluded that OTCMS who have worked for a considerable number of years should be trained and upgraded to sell antibiotics even if not all could be allowed:

“I have sold medicine for forty years and before that I was a pharmacy assistant for like three years whiles others have worked at the pharmacy shop for about five years before they got their licence. With this experience and additional training we will be able to do the work well” (IDI, OTCMS association, Brong Ahafo, respondent #2)

In contrast to the above, GHS participants and pharmacy council at the regional levels and AMR platform emphasized that OTCMS cannot be trained to dispense antibiotics because their level of education is inadequate.

“With their level of education, they are not qualified to dispense antibiotics so the law states clearly that they should only sell class-C (OTC) medicines which is okay” (IDI, pharmacy council, Brong Ahafo, respondent #1).

For CHPS facilities, GHS participants at the district level indicated that it is feasible to train health workers to dispense some antibiotics. It was underscored that health workers at the CHPS are already trained and doing similar activities. As such, they can be given additional training to dispense antibiotics.

“…the cadre of staff that we have at CHPS should be able to prescribe and dispense some antibiotics when given training. They are trained health staff who have been doing similar activities just that they do not have the exact training/guidelines to dispense antibiotics” (IDI, Municipal Health Directorate, Kintampo, respondent #2).

In contrast to those at the district level, GHS participants at the regional level and AMR platform were of the view that it is not feasible to train staff at CHPS to dispense antibiotics. It was stressed that care should be taken not to allow antibiotics to be dispensed at the CHPS just because we want to increase access. Participants rather suggested that the role of CHPS and OTCMS in fighting antibiotic resistance should be preventive rather than curative.

“CHPS and OTCMS role in preventing antibiotic resistance will be basically public health education because that is one of their core mandates; that is to educate the public on diseases, hygiene, and sanitation….This will contribute to education and advocacy on using medicines appropriately” (IDI with AMR platform members_respondent #2)

### Medical products

#### Whether OTCMS and CHPS require medical equipment to dispense antibiotic

Whilst some leaders of OTCMS association at the regional level mentioned that some medical equipment will be required to dispense antibiotics, others mentioned that there will be no need for such equipment or tools as they will not diagnose and prescribe, but only dispense with prescription from qualified health professionals.

“We will not prescribe antibiotics. We will get prescription from doctors before we dispense. We know that antibiotics are not to be taken anyhow…” (IDI, OTCMS association, Brong Ahafo, respondent #2)

For CHPS, participants of GHS at the district and regional levels acknowledged that a medical laboratory which is currently not part of CHPS with basic equipment may be required to do culture and sensitivity test. However, participants also suggested that rapid diagnostic test kits could help in diagnosis because the health system may not be able to equip the CHPS facilities with laboratories.

“…I don’t know whether we [health system] will be able to provide that [laboratory]. Also, looking at the personnel at the CHPS facilities, there are currently no laboratory technicians. As in the case of malaria, if we could have rapid diagnostic test kits for infectious diseases, then dispensing of antibiotics will be very effective” (IDI, Municipal Health Directorate, Kintampo, respondent #2)

#### Whether training OTCMS and CHPS to dispense antibiotics will affect the pricing

Two divergent perspectives emerged from the OTCMS association on whether training OTCMS to dispense antibiotics will affect the price of antibiotics. Whilst some mentioned that the prices of antibiotics will increase if they will have to buy equipment, i.e. rapid diagnostic test kits, others were of the view that prices will only increase if their suppliers increase the prices.

The price of antibiotics will increase if we would have to buy that equipment [rapid diagnostic test kit]; it will affect the price of the antibiotics but not very significant (IDI, OTCMS medicine sellers association, Kintampo, respondent #1)

For CHPS, GHS participants at the district level indicated that the prices of antibiotics may not be affected because prices of medicines were not determined at the CHPS facilities.

“…the CHPS are not responsible for the pricing because the pricing of each drug is determined nationally so basically, it wouldn’t affect it” (IDI, Municipal Health Directorate, Kintampo, respondent #2)

### Health finance

#### How OTCMSs and CHPS are funded

OTCMS are private entities funded by licensed holders or business owners. Medicines are either bought with cash or on credit and this is their only source of funding. OTCMS do not receive specific payments for providing health information to clients. CHPS are funded with money received from the national health insurance authority for services provided to insured patients. Non-insured patients pay for consultation and medicines. Health professionals at CHPS are paid by government. CHPS also receive donations from individuals and non-governmental organizations.

#### Perspective of possible financial challenges with incorporating antibiotic dispensing

Leadership of the OTCMS association at the district and regional level indicated that there will be no major financial challenge with including antibiotic dispensing as part of medicine OTCMS already dispense. This is because the pharmacy council does not force them to stock medicines they dispense. Also, they could buy medicines on credit from the pharmaceutical companies and pay later:

“Whenever new services are to be included, the pharmacy council just give you the training and OTCMS decide whether to buy the device/drug or not” (IDI, OTCMS association, Brong Ahofo, respondent #2).

However, they specified cost of training as the possible financial challenge which could affect efforts to train the OTCMS. It was emphasized that if the cost of training is moderate, OTCMS will largely attend. Absorbing the cost of training of OTCMS by government will be welcomed.

“In case the training fee is moderate, the OTCMS will pay and attend the training. For instance the pharmacy council training fee increased to GHS 100.00 [USD 17.34] this year (2019). Previously, it was GHS 50.00 [USD 8.67]” (IDI, OTCMS association, Brong Ahafo, respondent#2).

Possible financial challenge at the CHPS is the cost of organizing the training for the staff and monitoring/supervision after training by the district health directorate.

“Once training CHPS to dispense antibiotics becomes a policy... Bringing them together is money so it is going to be another challenge” (IDI, Municipal Health Directorate, Kintampo, respondent #3).

### Health information

#### Information required in training OTCMS and CHPS to dispense antibiotics

Leadership of the OTCMS at the regional level generally upheld that beyond training, a manual/guideline containing key information on antibiotics dispensing will be required for reference in daily practices. They further indicated that it is crucial that OTCMS are taught that antibiotics should be dispensed in full dose and on implications of not dispensing in full dose.

“it will be good to have a guideline for dispensing drugs [antibiotics]; sometimes if you are confused you can go through the book to help you know what to do” (IDI, OTCMS association, Kintampo, respondent #1).

For CHPS, GHS participants at the district level alluded that beyond antibiotics dispensing guidelines, health information materials such as posters and reporting format for antibiotics dispensed will be required.

“The CHPS has to be provided with health information materials such as posters, protocols, and reporting mechanisms/format so that they will be able to report on antibiotics dispensed” (IDI, Municipal Health Directorate, Kintampo, respondent #2).

#### Who should provide information required in training OTCMS and CHPS?

According to the leadership of OTCMS at the district and regional level, the pharmacy council supported by the FDA should be responsible for providing information for the training and dispensing of antibiotics by OTCMS and CHPS.

“Pharmacy council should provide us with information required to dispense antibiotics because they regulate OTCMS” (IDI, OTCMS association, Brong Ahafo, respondent #2).

With regard to CHPS, it was suggested that the GHS should lead in the training and provision of information for CHPS to dispense antibiotics.

“At the district level the medical superintendent should be the lead person in that training or maybe his/her representative because they are qualified clinicians and have the experience as well” (IDI, Municipal Health Directorate, Kintampo, respondent #2).

## Discussion

This study explored stakeholders’ perspectives on the feasibility of training OTCMS and CHPS facilities to dispense some antibiotics in Ghana. This study is important in the context where strategies for tackling antibiotic resistance tend to largely focus on top-down or expert-led approaches to scientific innovations, surveillance and antibiotic stewardship programmes[17]. In this study, we built on findings from our previous study in which suggestions emerged that OTCMS and CHPS could be trained to dispense some antibiotics to improve appropriate use at the community level [4]. This study therefore enabled us to bring to the attention of relevant stakeholders involved in pharmaceutical policy formulation and implementation in Ghana, the concerns of OTCMS and community members regarding the sale of antibiotics. In addition, the application of the WHO building blocks in designing the interview guides enabled us to broadly explore stakeholders’ perspectives on the health system dynamics which could influence the training of OTCMS and CHPS to dispense some categories of antibiotics. This further emphasized the need to be circumspect in considering emerging discussions around allowing OTCMS and CHPS to dispense some antibiotics as this will largely happen in a broader and existing health system framework.

Findings from this study show that as one moves from the district level through the region to the national level, the more the respondents disagreed with the suggestion to train OTCMS and CHPS to dispense some antibiotics. The fact that participants of GHS in the region and AMR platform at the national were not in support of antibiotics dispensing by OTCMS and CHPS facilities shows the need for further stakeholder engagement and dialogue as antibiotics are already dispensed by OTCMS and in CHPS facilities especially where there are midwives. Findings from our previous studies on the context of antibiotic access and use at the community level and the revised CHPS policy provides information that could guide the stakeholder engagements [4, 5, 7, 25]. As specified in the revised policy, district directors of health services may include midwifery services in the package of services for a specific CHPS and post a qualified resident midwife. Following this, midwives have been attached to some CHPS facilities to conduct deliveries [26]. In Ghana, according to the Health Professions Regulatory Body Act, 2013 (Act 857), midwives are eligible to prescribe registered antibiotics [27]. It is therefore important to consider the emerging suggestion to include antibiotic dispensing in the services of CHPS considering that some CHPS with midwives already dispense antibiotics if need be. Training CHPS could be a good starting point to improve appropriate antibiotic access and use as they are closer to community members and could reduce purchasing from OTCMS.

In the event that the emerging suggestion to train OTCMS and CHPS is considered, guidelines for dispensing/treatment will be an important tool, but supervision will equally be required to ensure adherence. Treatment guidelines are systematically developed statements that assist prescribers in deciding on appropriate treatment for specific clinical problems [28]. In Ghana, MoH has standard treatment guidelines for prescribers which reflects the consensus on optimal treatment options within the health system[29, 30]. Similar guidelines could be developed for OTCMS. On the supervision, this finding further reiterates the need to strengthen the implementation of pharmaceutical regulations especially when some stakeholders disagree with the suggestions to train OTCMS to dispense some of antibiotics. This is important because currently antibiotics can easily be purchased with or without prescription from the OTCMS and even drug peddlers [4, 5]. Without enforcing the regulations, OTCMS will continue to sell antibiotics with the aim of providing health services to the community whilst making profit [4]. The overarching concern therefore remains how to engage OTCMS to support the fight against antibiotic resistance in the context where regulating or supervising the activities is still a challenge.

It is therefore crucial for relevant stakeholders specially the Ghana AMR platform to strategically engage the OTCMS through the leadership of their association to discuss how best to include them in their activities to combat AMR. The OTCMS association with branches in the districts and regions coordinates the activities of OTCMS. These are important stakeholders who contribute to ensuring good pharmaceutical practice or otherwise [23]. OTCMS could therefore be engaged to support public health education against inappropriate antibiotic use taking cognizance of how to ameliorate the effect on their business. The starting point could be the inclusion of their representatives on the AMR platform which is currently implementing various activities towards the containment of AMR in Ghana. Once OTCMS feel part of the system, they could be incentivized to contribute to fighting antibiotic resistance. OTCMS are widely distributed across Ghana and are potentially an important ally in tackling AMR by improving antibiotic use through better information. As indicated earlier, this should be seen in the context where the regulator (i.e. the Pharmacy Council) is inadequately resourced to be able to effectively supervise and enforce the regulations on the sales of antibiotics [4].

The lack of laboratories in CHPS to enable them to effectively dispense antibiotics also emerged as a possible challenge. This task shifting concept could be feasible considering the emergence of rapid diagnostic tests or point-of-care tests for infectious diseases [31]. Rapid diagnostic test kits for testing gastrointestinal, respiratory and sexually transmitted infection have shown promise in reducing unnecessary antibiotic use [32, 33]. CHPS could be equipped with these innovative assays to provide better care and improve antibiotic dispensing and also meet the high emerging demand of the community for curative services provision in the CHPS facilities [25]. This will reorient community members to seek care at CHPS instead of OTCMS as equipping healthcare providers at community level will improve dispensing of antimicrobials [34]. Clients could pay for only the test if it is negative as antibiotics may not be required.

## Recommendation

The GHS, MoH, and Pharmacy council are encouraged to engage with representation from OTCMS and CHPS for further discussion with relevant stakeholders as part of efforts to improve appropriate antibiotic access and use at the community level. Meanwhile, the GHS and MoH should consider massive extension of approved health facilities and services to rural communities to enhance appropriate access and use of antibiotics. Furthermore, some of the CHPS facilities could be upgraded into health centres with qualified physician assistance who could prescribe some basic antibiotics and refer when need be as has been done with midwives. The GHS, MoH and pharmacy council should also consider providing incentives to qualified pharmacist to set up pharmacy shops in rural communities as they are underrepresented. Community members who need antibiotics could be served appropriately if they have prescriptions or be given appropriate advice and referral to approved health facilities.

## Strengths and limitations

The interview guide was situated in the six health system building blocks of the WHO to explore stakeholders’ perspectives on the feasibility of dispensing antibiotics. This enabled us to explore stakeholders’ perspectives on various aspect of the health system. Some components of the guide could not be explored among members of the Ghana AMR platform during discussions on the platform because their initial comments and responses indicated that they were not entirely in support of suggestions to train OTCMS and CHPS to dispense antibiotics. However, we conducted a follow-up IDIs to explore their perspectives on the role of OTCMS and CHPS in ensuring that antibiotics are accessed and dispensed appropriately at the community level.

## Conclusion

There was a general lack of consensus among stakeholders on whether OTCMS and CHPS should be trained and allowed to dispense some antibiotics. This requires further stakeholder engagement to interrogate the differences in perspectives and devise how stakeholders could work together to improve antibiotic access and use at the community level. This is in line with the strategic objective of the national AMR policy which seeks to engage all relevant stakeholders in efforts to improve antibiotic access and use.

## Data Availability

The datasets used and/or analysed during the current study are available from the corresponding author on reasonable request.

## Appendix 1

**Table. 3:** Information on stakeholder institutions/organization

The Ministry of Health formulates health policies, sets standards for the delivery of health care and provides strategic direction for health delivery services in Ghana [26]. Ghana health service is responsible for the implementation of national health policies. It provides health service with special emphasis on primary health care at regional, district and sub-district levels in accordance with national policies [35]. The pharmacy council is a statutory regulatory body responsible for securing the highest standards in the practice of pharmacy in the country [36]. Food and drugs authority is responsible for protecting public health by assuring the safety, efficacy and security of human and veterinary drugs, food, biological products, cosmetics, medical devices, household chemical substances, tobacco and the conduct of clinical trials in Ghana [37]. The Over the counter medicine sellers association with branches in the districts and regions coordinates the activities of chemical sellers across GhanaThe AMR working group is a platform created by the Ministry of Health to develop AMR policy for Ghana to address all AMR-related issues from a “One health” approach [38]. The AMR Platform is a multi-sectorial working group including representatives from human, animal (livestock, fish, poultry, etc.), plant and the environment. Representatives of institutions and agencies represented on the Ghana AMR platform include Ministry of Health, Ghana Health Service, Food and Drug Authority, Ministry of Food and Agriculture, Ministry of environment science technology and innovation, University of Ghana (Department of Microbiology and Department of Veterinary medicine), etc	

## Abbreviations

LMIC: Low and middle income counties
CHP: Community Health Post
OTCMS: Over the counter medicine sellers
CHPS: Community-based Health Planning and Services
AMR: Antimicrobial resistance
FGD: Focus group discussions
IDI: In-depth interviews
KNM: Kintampo North Municipality
GHS: Ghana Health Service
MoH: Ministry of Health
FDA: Food Drugs Authority
